# Role of Oxidative Stress in the Mechanisms of Anthracycline-Induced Cardiotoxicity: Effects of Preventive Strategies

**DOI:** 10.1155/2021/8863789

**Published:** 2021-01-25

**Authors:** Rodrigo Carrasco, Rodrigo L. Castillo, Juan G. Gormaz, Montserrat Carrillo, Paaladinesh Thavendiranathan

**Affiliations:** ^1^Division of Cardiology, Peter Munk Cardiac Centre and the Ted Rogers Centre for Heart Research, University Health Network, Toronto, Ontario, Canada; ^2^Medicine Department, East Division, Faculty of Medicine, University of Chile. Santiago, Chile; Critical Care Patient Unit, Hospital Salvador, Santiago, Chile; ^3^Faculty of Medicine, University of Chile, Santiago, Chile

## Abstract

Anthracycline-induced cardiotoxicity (AIC) persists as a significant cause of morbidity and mortality in cancer survivors. Although many protective strategies have been evaluated, cardiotoxicity remains an ongoing threat. The mechanisms of AIC remain unclear; however, several pathways have been proposed, suggesting a multifactorial origin. When the central role of topoisomerase 2*β* in the pathophysiology of AIC was described some years ago, the classical reactive oxygen species (ROS) hypothesis shifted to a secondary position. However, new insights have reemphasized the importance of the role of oxidative stress-mediated signaling as a common pathway and a critical modulator of the different mechanisms involved in AIC. A better understanding of the mechanisms of cardiotoxicity is crucial for the development of treatment strategies. It has been suggested that the available therapeutic interventions for AIC could act on the modulation of oxidative balance, leading to a reduction in oxidative stress injury. These indirect antioxidant effects make them an option for the primary prevention of AIC. In this review, our objective is to provide an update of the accumulated knowledge on the role of oxidative stress in AIC and the modulation of the redox balance by potential preventive strategies.

## 1. Introduction

Over the past two decades, there have been significant improvements in the early detection and pharmacological treatment of cancer, leading to a dramatic increase in survivorship [1] [2]. However, this improvement in the life expectancy of cancer patients has also led to an increase in the pool of patients at risk of experiencing long-term chemotherapy-related side effects. Chemotherapy-induced cardiotoxicity is a common complication of many cancer therapeutics and a frequent cause of morbidity and mortality in cancer survivors. Amongst cancer therapeutics, anthracycline compounds contribute to a significant proportion of the cardiovascular disease burden.

Anthracyclines are specific cytostatic antibiotics, and they represent one of the most used chemotherapeutic agents to treat many solid cancer tumors and hematological malignancies [3]. Its clinical use has, however, been limited by the development of cardiotoxicity in a cumulative dose-dependent manner [4]. This side effect impacts the long-term prognosis of patients treated successfully from an oncological point of view [5]. The effects of anthracycline-induced cardiotoxicity (AIC) have become more apparent for several reasons, including a stricter clinical follow-up and improvements in cardiovascular diagnostic methods [6].

A meta-analysis by Lotrionte et al. evaluated the late incidence of AIC after a median of 9 years follow-up, finding an occurrence of clinically evident cardiotoxicity in 6% and subclinical cardiotoxicity in 18% [7]. More recently, Cardinale et al. prospectively followed adult patients treated with anthracyclines and found an incidence of AIC of 9%. AIC was detected within the first year after completion of treatment in 98% of cases [8]. An important finding was that close monitoring of cardiac function during this period allowed early detection and treatment of cardiotoxicity, with significant left ventricular ejection fraction (LVEF) recovery in most cases [8]. Current clinical interventions are focused on prompt detection of subclinical damage through cardiac imaging and biomarker techniques; however, these interventions are focused on damage control rather than a preventative approach. Unfortunately, despite decades of research efforts to improve clinical strategies of primary prevention of AIC, there is still no satisfactory therapy to avoid this complication. Therefore, a better understanding of the mechanisms of cardiotoxicity could offer new opportunities to provide optimal primary prevention strategies.

The objective of this review is to provide an update on the accumulated knowledge regarding the early and critical role of oxidative stress in the damage mechanisms of AIC and discuss the potential benefits of preventive strategies that reduce oxidative stress damage through lifestyle changes, physical exercise, and pharmacological therapies to reduce risk factors and environmental stressors.

## 2. Mechanisms of Anthracycline-Induced Cardiotoxicity

Anthracyclines tend to accumulate in the mitochondria, which partly explains their tendency to accumulate in myocardial tissue, which is characterized by a high mitochondrial density due to its high energetic demand [9]. Specifically, in the heart, cardiomyocytes have classically been considered the primary cellular target of the toxic anthracycline effect. However, other cell types such as cardiac progenitor cells, cardiac fibroblasts, and endothelial cells have also been recognized as potential anthracycline targets, which through paracrine effects mediated by microRNA (miRNA) and other cells signals could also be involved in cardiomyocyte injury [10, 11]. Several pathways have been proposed to explain the development of AIC, such as the potential generation of oxidative stress, inhibition of topoisomerase 2*β* (Top2*β*), changes in iron metabolism, and Ca2+ signaling [10]. However, the precise reason as to why only some patients develop AIC remains unclear, suggesting a multifactorial origin that could comprise complex interactions between the different involved pathways [12].

### 2.1. Anthracycline Accumulation in the Heart

One of the most significant determinants of the development of AIC is the cumulative dose of anthracyclines in cardiac tissue [13], which is also related to the magnitude of redox imbalance. For this reason, the cumulative dose remains the leading risk factor for AIC [7]. Furthermore, anthracyclines are more retained within cardiomyocytes than in cells of other noncardiac tissues [14]. The primary process that determines heart accumulation is the liver biotransformation to secondary alcohol metabolites. These metabolites are more polar than original compounds and exhibit a higher entry rate and reduced elimination rate from cardiac tissue [15, 16]. Doxorubicinol, the most crucial alcohol metabolite of doxorubicin, has been implicated in the cardiotoxicity observed in doxorubicin-treated patients [17, 18]. The NADPH-dependent reduction of doxorubicin to doxorubicinol is catalyzed by carbonyl reductase 1 (CBR1), a well-characterized monomeric enzyme present at high basal levels in the liver [19] and carbonyl reductase 3 (CBR3) less characterized, present in the liver at low basal levels [20]. Animal models have shown that high metabolizer mice develop an accelerated cardiotoxicity course through an increased heart accumulation of secondary alcohol metabolites [21]. Therefore, hepatic biotransformation of anthracyclines represents a potential research focus to establish higher risk groups and new targets for pharmacological interventions. At a subcellular level, the accumulation of anthracycline secondary alcohol metabolites in cardiomyocytes is especially pronounced in the mitochondria, affecting the mitochondrial transmembrane potential, and inhibiting the complex I respiratory chain, which causes an impairment of mitochondrial metabolism and subsequent mitochondrial dysfunction [22].

### 2.2. Convergence of “Redox Cycling” and “Topoisomerase 2*β*” Hypotheses: ROS Generation and AIC

Despite the multiple mechanisms of AIC described, there is consensus in considering “Redox Cycling” and the “Top2*β* inhibition” as the two main mechanisms. Several preclinical and clinical trials, as well as genetic studies, have shown that oxidative stress generated by reactive oxygen species (ROS) accumulation is the crucial step in the development and progression of AIC [23–26]. Initially, it was suggested that early ROS would be produced by a direct anthracycline interaction with the mitochondrial electron transport chain [9] [27]. The enzymatic antioxidant defences are lower in the cardiac tissue compared with other organs (liver and kidney); this can make the heart particularly vulnerable to free radical damage [12].

Later studies suggested that interaction with topoisomerase 2*β* (Top2*β*) would be the initiating trigger for cardiotoxicity [24]. Top2*β* is an enzyme involved in nuclear and mitochondrial DNA replication, which plays a crucial role in AIC for the formation of anthracycline–DNA-topoisomerase 2*β* complexes [24]. Some mechanisms studied as possible mediators of Top2*β*-dependent cardiotoxicity include p53 activation, modulation of peroxisome proliferator-activated receptor *γ* coactivator-1*α* (PGC-1*α*) and -1*β* (PGC-1*β*), and modulation of antioxidant enzyme gene transcription [24]. Both PGC-1*α* and PGC-1*β* are highly expressed in the heart, playing a critical role in mitochondrial biogenesis regulating oxidative metabolism [28, 29]. They have also been associated with heart failure pathogenesis [30, 31]. Top2*β* inactivation by anthracycline accumulation heavily reduced the expression of PGC-1*α* and PGC-1*β* in rat cardiomyocytes [24]. Nevertheless, the same data also indicate that anthracycline interaction with Top2*β* leads to mitochondrial dysfunction with the subsequent generation of ROS-mediated oxidative stress [24]. This triggers a progressive disruption in Ca+2 homeostasis, inflammation, and the inhibition of ATP generation [32], which in turn promotes apoptosis and cardiac remodeling, critical events in the development of AIC [24, 33].

According to the time of presentation, AIC was previously classified as acute, subacute, or chronic. Recent findings challenge this old classification, suggesting that AIC is a continuous phenomenon, starting at the myocardial subcellular level, followed by a progressive functional decline, which could lead to overt heart failure [10]. In all patients who develop AIC, the damage associated with oxidative stress starts very early at the molecular level, which causes subcellular dysfunction through various mechanisms [34]. If antioxidant defences are rapidly overwhelmed, prompt damage could occur, leading to a rare short-term event of acute AIC [34]. These acute AIC events are infrequent but associated with high mortality risk, and their manifestations may include pericarditis, arrhythmias, and left ventricular systolic dysfunction (LVSD) [35] [13]. However, it should be noted that the majority of patients with AIC do not manifest a clinically evident acute cardiotoxicity; they are usually asymptomatic and present some signs of acute subclinical cardiotoxicity evidenced exclusively by functional alterations in the left ventricle (changes in longitudinal strain or LVEF) or by an increase in cardiac biomarkers [36, 37].

### 2.3. - Mediators of Increased Susceptibility to Oxidative Stress Injury and AIC

#### 2.3.1. Overview of Oxidative Stress in the Cardiovascular System

Since oxidative stress plays a crucial role in AIC, it is important to understand how ROS are generated and countered in the cardiovascular system. Oxidative stress can be defined as an imbalance between the generation and detoxification of ROS [38]. At physiological levels, a slight increase in reactive oxygen species (ROS) could induce protective effects through triggering redox signaling, for example, via improving adaptive antioxidative response by activating of Keap1/Nrf2/ARE pathway. If the ROS generation outweighs the antioxidative capacity, then at higher ROS levels, cell damage and endothelial dysfunction arise contributing to the development of atherosclerosis and heart injury [39].

Aging, genetic predisposition, traditional cardiovascular risk factors, and environmental factors can induce oxidative stress, particularly in the heart and vessels [39]. At the cardiac cellular level, enzymatic sources for ROS, such as the nicotinamide adenine dinucleotide phosphate (NADPH) oxidases (NOX), uncoupled nitric oxide (NO) synthase, and mitochondria, are all considered relevant sources of ROS that contribute to the development of vascular and cardiac dysfunction [38, 39]. Importantly, mitochondria amplify ROS derived from NOX and may thereby function as a “redox hub” in cardiac pathophysiology [40]. ROS determines myocardial calcium (Ca2+) overload, an event that plays a causal role in contractile dysfunction, arrhythmias, and the maladaptive cardiac remodeling process by inducing hypertrophic signaling, apoptosis, necrosis, and autophagy [41].

#### 2.3.2. Cardiovascular Risk Factors Are Associated with a Significant Susceptibility to Oxidative Stress Injury

The pathophysiological effects of traditional cardiovascular risk factors on the cardiovascular system are driven by oxidative stress. Therefore, it is not surprising that many of the risk factors for AIC such as age > 65 years, valvular heart disease, baseline left ventricular dysfunction, arterial hypertension, African-American ancestry, renal failure, concomitant exposure to radiation and/or trastuzumab, iron overload, and genetic factors [3] [42] have oxidative stress as a common element. Abdel-Qadir et al. developed and validated a multivariable risk prediction model for major adverse cardiovascular events (MACE) in patients with early-stage breast cancer, where age, hypertension, diabetes, ischemic heart disease, atrial fibrillation, HF, cerebrovascular disease, peripheral vascular disease, chronic obstructive pulmonary disease, and chronic kidney disease were significantly associated with MACE [43]. Again, many of the risk factors for MACE in this study have oxidative stress as a common pathophysiological mechanism for cardiovascular disease and hence increase susceptibility to AIC.

#### 2.3.3. Genetic Susceptibility to Oxidative Stress Injury

Susceptibility due to inherited genetic, variation could partially explain the high interindividual variability in risk of AIC [44, 45]. In this sense, a genetic approach has been used to identify patients at increased risk. Several polymorphisms in candidate genes have been proposed, some of them relate to the accumulation or biotransformation of anthracyclines, but most of them related to redox balance, either associated with antioxidant defence or ROS generation [44]. Some of these candidate genes are CBR1 and 3, NAD(P)H quinone oxidoreductase, which have been the most described and are discussed in more detail below, and also glutathione S-transferase and multidrug resistance proteins 1 and 2 [46].

*(1) Carbonyl Reductase 1 and 3*. As previously described, CBR1 and CBR3 catalyze the NADPH-dependent reduction of doxorubicin to doxorubicinol. Therefore, polymorphisms that contribute to higher levels of hepatic transformation result in a greater accumulation of toxic metabolites within the heart [21, 47]. Preliminary epidemiological data have shown that the human CBR3 polymorphisms, but not CBR1, are associated with differential cardiac outcomes in doxorubicin-treated patients [47, 48]. For example, in childhood cancer patients treated with doxorubicin, a relatively common polymorphism in the CBR3 gene (present in 30% of Caucasians) that encodes for a nonsynonymous amino acid change (V244M) was associated with a decreased risk of developing cardiomyopathy [47]. Furthermore, another CBR3 variant (11 *G* > *A*) has been shown to influence the relative expression of CBR3—and subsequent doxorubicinol formation—in a cohort of Southeast Asian breast cancer patients [48].

*(2) NADPH Oxidase (NOX)*. NOX has been suggested as one of the most important sources of ROS in the cardiovascular system. Five NOX isoforms have been identified; in particular, NOX2 and NOX4 play a significant role in the heart signaling, as they are bound to the sarcolemma of the cardiomyocytes [49]. Due to the importance of NOX in ROS generation, a possible association of some of their polymorphisms with the development of AIC has been studied. Thus, individuals with less active genetic variants of NOX might be protected from heart damage and fibrosis induced by anthracyclines [49].

This hypothesis has been supported by a case-control clinical study of doxorubicin-induced cardiotoxicity, showing that NADPH genetic variations can modulate the risk for acute and chronic cardiac events [50]. Also, another study confirmed the predictive value of the three NADPH oxidase polymorphisms (rs1883112, rs4673, and rs13058338), although only one of them (rs1883112) was significant in the multivariable analysis [51]. Finally, the role for the genetic variants in the generation of cardiac lesions was demonstrated in a retrospective case-control study that evaluated cardiac histological lesions and three different NADPH genotypes (rs1883112, rs4673, and rs13058338) in 97 consecutive decedent patients with cancer diagnosis (48 treated with anthracyclines) [52]. One polymorphism of the subunit p40phox of NADPH oxidase was strongly associated with increased myocardial interstitial fibrosis, which could be explained by the higher level of the regulatory subunit p40 in NOX2. On the other hand, other polymorphisms were associated with a lesser degree of oxidative stress, apoptosis, and myocardial damage after anthracycline treatment, which might be explained by lower activity or expression levels of NOX2 and NOX4. Thus, the findings of this study provide a possible mechanistic link between NADPH functional SNPs and cardiac dysfunction [52].

*(3) Other Candidate Genes*. A meta-analysis assessed the role of genetic polymorphisms in AIC based on 28 studies examining 84 different genes. This analysis revealed that polymorphisms in three genes were significantly associated with an increased odds of cardiotoxicity in individuals treated with anthracyclines, and two of them were associated with oxidative stress: CYBA and RAC2 genes [53]. Genetic variants in CYBA altered the NADH/NADPH oxidase activity and may be associated with the excessive production of ROS [54]. Rac2, encoded by the RAC2 gene, is a mitochondrial protein that is required in the electron transfer reaction of NADPH oxidase during the formation of ROS [55]. Alteration of the RAC2 gene results in mitochondrial dysfunction and, thus, an increase in ROS production [53]. Despite all the existing data, we must emphasize that the individual risk provided by these candidate genes was only moderate, so new prospective studies are still needed in order to validate these genetic biomarkers for clinical application [53]. Therefore, currently, the potential role of these genes for a pharmacogenomic screening approach in routine clinical practice before anthracyclines therapy remains limited.

### 2.4. Second-Hit Hypothesis

The second-hit hypothesis suggests that the ability of the heart to adapt to new stress conditions is impaired after exposure to anthracyclines [56]. This means that the cardiac tissue of patients previously treated with anthracyclines, even with no previous evidence of measurable subclinical damage, may have a decreased resistance to new injuries, resulting in an increased risk of developing heart failure [57]. Progenitor cell impairment secondary to anthracyclines, concomitant or subsequent treatments with other antineoplastic drugs, and genetic predisposition may play a role in the mechanism to explain the second-hit hypothesis [56]. However, the second-hit events are more likely to relate to the development of new pathological conditions, mainly cardiovascular risk factors that are associated with an oxidative imbalance, for example, hypertension, diabetes mellitus, obesity, or atrial fibrillation [58]. Furthermore, the development of coronary artery disease has also been found to be related with the late development of left ventricular dysfunction in patients treated with anthracyclines [8].

From a clinical point of view, as cancer survivors are at higher risk for other noncommunicable diseases [59], second-hits will not be infrequent in anthracycline-treated patients. It is important to highlight that cardiovascular risk factors could contribute as much as a cancer treatment to the development of diastolic and systolic dysfunction in childhood cancer survivors [60]. For instance, survivors with metabolic syndrome are more likely to have abnormal longitudinal strain and diastolic dysfunction [61]. Survivors also have a higher prevalence and a more premature presentation of hypertension and dyslipidemia [62]. In fact, it has been described that childhood cancer survivors are 15 times more likely to develop congestive heart failure and 10 times more likely to have coronary artery disease compared to their siblings [63]. Cardiovascular risk factors are known to be more frequent in survivors of breast, prostate, colorectal, and gynaecologic cancers compared to age-matched individuals, with a reported prevalence of overweight/obesity, diabetes, and hypertension of 62%, 21%, and 55%, respectively [64]. A recent study showed that older age (>60 years) or preexisting chronic diseases like hypertension and diabetes were present in the majority of patients with heart failure hospital presentations after the diagnosis of early-stage breast cancer [65], again emphasizing the importance of concomitant cardiovascular risk factors. Therefore, the development of adequate surveillance follow-up programs in cancer survivors to promote a healthy lifestyle and the early detection, assessment, and management of cardiovascular risk factors are essential [66].

## 3. Effects of Cardioprotective Strategies on the Redox Balance for the Prevention of Anthracycline-Induced Cardiotoxicity

### 3.1. Overview of Antioxidant Strategies in Cardiovascular Diseases

For many decades, researchers have tried to elucidate the role of oxidative stress in cardiovascular disease, establishing that a redox imbalance with subsequent oxidative stress is essential for the development of many cardiovascular diseases, including atherosclerosis, hypertension, and congestive heart failure [67]. However, a wide variety of antioxidant strategies to prevent cardiovascular disease has not yielded positive results with respect to their clinical efficacy [68–70].

Multiple reasons could explain these poor results, such as inadequate choice of drugs, dosage, and duration of antioxidants interventions [67, 71–73]. All this could be summarized by stating that the strategies that have been studied may be too simple by trying to restore the redox balance, generally using antioxidants with direct effects [52–54] [48], instead of strategies with indirect antioxidants, which have better preclinical evidence of effectiveness [55]. To better understand this, it is also essential to recognize that there are two types of small-molecule antioxidants which provide cellular protection against oxidative stress: (i) direct antioxidants, which are redox-active, short-lived because they are sacrificed during the process of their antioxidant actions and need to be replenished or regenerated, and may evoke prooxidant effects; and (ii) indirect antioxidants, which activate the Keap1/Nrf2/ARE pathway resulting in transcriptional induction of a battery of phase 2 enzymes, that act catalytically, are not consumed, have long half-lives, and are unlikely to evoke prooxidant effects [74]. Another factor could be the influence of the genetic factors previously explained, which raises the possibility that only some clusters of patients will benefit from antioxidant treatment [75].

### 3.2. Statins

3-Hydroxy-3-methylglutaryl coenzyme A reductase (HMG-CoA reductase) inhibitors are well known for their lipid-lowering capacity, but also their anti-inflammatory and pleiotropic antioxidant effects. Treatment with statins has been proposed as an option for primary prevention in the setting of anthracycline-induced cardiomyopathy. In the clinical setting, a previous study that included 40 patients undergoing anthracycline therapy randomized them to receive statin therapy versus placebo for six months. The decrease in the mean left ventricular ejection fraction after the completion of treatment was significant in the control group as compared with the statin group, and the mean increase in left ventricular end-diastolic diameter and left ventricular end-systolic diameter was significantly lower in the statin group as compared with controls [76]. An observational cohort study of breast cancer patients showed that uninterrupted statin use during anthracycline chemotherapy was associated with a significantly lower risk of incident heart failure [77].

Previous studies in animal models have demonstrated the cardioprotective effects of statins in anthracycline-induced cardiomyopathy, and they have also allowed a better knowledge of the intracellular pathways involved that explain this effect [78].

From a mechanistic point of view, statins have been shown to reduce the doxorubicin-induced cardiac inflammatory response and oxidative stress and to attenuate mitochondrial apoptotic pathways in animal models [79, 80]. However, we must consider that several of the pleiotropic effects of statins to prevent AIC are mediated by a reduction of oxidative injury. First, statins have been found to preserve mitochondrial membrane potential in response to oxidative stress. This effect could be mediated by NO, activating mitochondrial ATP-sensitive potassium channels (mitoK_ATP_), which results in cardioprotection [81]. Second, Riad et al. investigated the cardioprotective effects of fluvastatin in doxorubicin-induced cardiomyopathy in a mouse model. In this study, statin treatment improved cardiac function, associated with an increase of the expression of the antioxidative enzyme SOD2 and secondarily decreasing tumor necrosis factor *α* (TNF-*α*) levels, suppressing doxorubicin-induced overexpression of the proapoptotic protein Bax, decreasing cardiac nitrotyrosine production, and activated mitochondrial-located antioxidative and antiapoptotic mechanisms [80].

Another mechanism by which statins have shown efficacy in AIC prevention is through downstream inhibition of Rac1 [79, 82]; however, this effect is also related to an antioxidant effect. Cholesterol-independent cardioprotective effects of statins have been traced back to the inhibition of Rho GTPase Rac1 signaling [83]. At the same time, the known antioxidative effects of statins [84] could be explained by this inhibition of Rac1, which could lead to a reduced intrinsic generation of ROS since Rac1 regulates the NADPH oxidase complex [85].

Pharmacological characteristics of statins, such as a fixed dosage and lack of hemodynamic effects, make them an attractive option for primary cardiotoxicity prevention [86]. Ongoing prospective randomized studies are investigating the potential role of statins in the primary prevention of anthracycline-induced cardiomyopathy.

### 3.3. ACE Inhibitors and Aldosterone Antagonists

Angiotensin-converting enzyme inhibitors (ACEi) and angiotensin II receptor blockers (ARBs) are part of the standard pharmacologic therapy used in patients with heart failure and reduced left ventricular ejection fraction (HFrEF) due to their well-proven effect on cardiac remodeling and, consequently, mortality reduction in this population [87, 88]. The potential effect of ACE inhibitors in the treatment of AIC patients was first demonstrated more than twenty years ago by Jensen et al. in a small observational study of patients with AIC [89]. Subsequently, secondary prevention with ACE inhibitors is now well established, with early detection and treatment of cardiotoxicity results in at least partial LVEF recovery in most cases [8].

The evidence for primary prevention of AIC with these agents is mainly supported by small observational studies and single-center randomized clinical trials [90]. ACE inhibitors are often used in combination with other interventions (such as beta-blockers), and there are only a few clinical trials specifically designed to study the use of an ACE inhibitor alone (enalapril) for AIC prevention [91, 92]. Evaluation of the evidence in two meta-analyses has suggested a potential role as a prophylactic intervention [90], and that neurohormonal therapies in single or combination strategies are associated with higher LVEF in follow-up, although absolute changes in LVEF are small and could be within intertest variability for the LVEF measurement [93]. However, recently, a multicenter randomized trial (ICOS-One Trial) compared two strategies for the prevention of AIC with enalapril: primary prevention versus a biomarker-guided strategy during treatment with anthracyclines [94]. No differences were found between primary prevention with enalapril versus treatment with enalapril guided by early detection of troponin elevation [94]. These results suggest that primary prevention with enalapril would not be superior to early treatment with enalapril when subclinical damage (elevation of biomarkers) is detected [94].

Aldosterone antagonists act by blocking the final step of the renin-angiotensin-aldosterone system in different organs. Several in vitro, preclinical, and clinical studies have established the importance of this target in heart failure and cardiac remodeling [95–97]. In the original report, Jensen et al. reported that spironolactone could enhance the effects of ACE inhibitors in AIC treatment [89]. However, this study encouraged the use of these two clinical interventions simultaneously to ensure the prevention of AIC [89]. The evidence on using aldosterone antagonist alone (spironolactone and eplerenone) is limited [10].

Considering the scarce evidence for aldosterone alone for AIC prevention and that the most robust evidence among ACE inhibitors is associated with enalapril [91, 92], which has shown an *in-vivo* capacity to inhibit oxidative stress, one could deduce that an antioxidant effect might be involved beyond the block of the renin-angiotensin-aldosterone axis [98, 99] [100]. From a redox point of view, we could also hypothesize a potential synergistic mechanism between enalapril and spironolactone, associated with two independent potential antioxidant mechanisms more than due to a specific receptor-mediated response. According to basic and preclinical studies, the spironolactone antioxidant effects have been associated with NADPH oxidase inhibition [101, 102], and enalapril oxidative stress abrogation has been associated with an enhancement of intracellular antioxidant defences (glutathione GSH-dependent antioxidant defences) [99, 100].

### 3.4. Beta-Blockers with Antioxidant Properties: Carvedilol and Nebivolol

Beta-blockers promote autonomic and neurohormonal regulation in the presence of cardiac dysfunction, leading to a positive impact on the cardiac remodeling of the left ventricle, resulting in reduced mortality from heart failure [88, 103]. With respect to the properties of the different beta-blockers, only carvedilol and nebivolol have antioxidant effects, potentially giving them some comparative advantages over other beta-blockers.

#### 3.4.1. Carvedilol

Although all beta-blockers could have a preventive effect in AIC, carvedilol has been one of the most studied in this setting [93]. Its potent antioxidant property distinguishes it from other *β* adrenergic receptor antagonists [104]. In this sense, carvedilol is superior to atenolol (which represents an antagonist of *β* adrenergic receptors, but without antioxidant properties) in reducing the negative impact induced by doxorubicin in systolic function, as well as the increase in lipoperoxidation (a product of oxidative stress injury in biological membranes) [105]. Carvedilol is a *β*-blocker with unique ROS-suppressive properties, even at subtherapeutic doses [106]. There is still uncertainty about its clinical benefit in primary prevention of AIC, based on mixed results from clinical [107–110] [111] and observational studies [112]. The CECCY trial, the most contemporary clinical study, was a randomized, double-blind, placebo-controlled protocol of carvedilol in 200 anthracycline-treated women with HER2 negative breast cancer. This study failed to prevent a ≥10% reduction in LVEF at six months. Nevertheless, in that protocol, carvedilol was able to prevent other manifestations of cardiotoxicity, reducing the number of patients experiencing increases in serum Troponin I (TnI) levels and attenuating its peak levels. There was also a trend towards a lower increase in left ventricular diastolic diameter and a reduction in the percentage of patients with diastolic dysfunction [111]. *In vitro* studies in cardiomyocytes have suggested that the cardioprotective effect of carvedilol is driven by its antioxidant properties [113], which have also been suggested after some clinical trials conducted [114], and as other *β*-blockers evaluated have not shown such a significant attenuation of AIC in clinical settings [115].

The variability of carvedilol in AIC prevention and its magnitude between different studies could be explained by intrinsic and extrinsic factors [93].

Carvedilol's antioxidative properties could be associated with its effects against mitochondrial dysfunction, which is one of the mechanisms associated with AIC and is characterized by a secondary ROS generation [116]. The specific mechanism to prevent mitochondrial dysfunction could be mediated by the stimulation of mitochondrial biogenesis by carvedilol, which results in a functional gain of the mitochondria [116]. Finally, increased expression of PGC-1*α* and mitochondrial biogenesis induced by carvedilol might suggest a new mechanism of the therapeutic effects of carvedilol in heart failure and AIC [116].

Therefore, it is currently unclear whether the potential protective effect of carvedilol is due to its antioxidant activity and reduction in lipid peroxidation or whether it is due to its *β*-blocker properties [117].

Interestingly, antioxidants at standard oral doses are not able to induce enough local heart effects to appreciate clinical benefits in cardiac conditions associated with oxidative stress. However, since carvedilol has an affinity for cardiac tissue, it can show local effects that are impossible to appreciate with generic antioxidants, but even this appears to be insufficient to prevent AIC. Nonintrinsic carvedilol factors could include a variable cumulative dose of anthracycline within the different studies, high individual variability in anthracyclines bioavailability [48], population heterogeneity, differences in risk factors profiles [7], and variability of chemotherapy protocols. These factors can determine the expected AIC incidences for a particular study and, therefore, determine a greater or lesser carvedilol efficacy in that protocol when compared to groups of patients with a standard risk. It is expected that populations with higher incidences of AIC are more prone to benefit from cardioprotective interventions than lower-risk populations.

#### 3.4.2. Nebivolol

Nebivolol is a highly selective *β*1 receptor beta-blocker drug, which is approximately 3.5 times more *β*1-selective than bisoprolol [118]. Unlike other beta-blockers with vasodilator effects (carvedilol and labetalol), which are mediated by blocking alpha-adrenergic receptors, nebivolol induces nitric oxide-dependent vasodilation, mediated by its agonist effect on endothelial *β*3 receptors that stimulate the enzyme nitric oxide synthase [119–121] and activate the NO/cGMP/PKG signaling pathway [122]. These endothelium-dependent vasodilation properties have been associated with a more significant blood pressure reduction in mechanistic studies than other beta-blockers [117]. Nebivolol is also characterized by antiproliferative, anti-inflammatory, and antioxidant properties, which would give additional value to its already indicated antihypertensive and endothelial effect [123]. At the subcellular level, the increase in nitric oxide formation following treatment with nebivolol has shown to increase cytosolic free zinc at the cardiomyocytes, with inhibition of intracellular and mitochondrial calcium overload and consequent protection against the effects of ROS and lipid peroxidation involved in hypertensive heart disease [124, 125].

With respect to the potential properties of nebivolol to prevent AIC, experimental studies in rats have shown antiapoptotic effects on cardiomyocytes and a reduction of ventricular dysfunction [126]. An antioxidant reinforcing effect would be involved as one of the protective mechanisms after treatment with nebivolol, which would be explained by an increase in the activities of glutathione peroxidase and Mn-superoxide dismutase, in addition to a release of nitrite/nitrate in cardiac tissue, which has been evidenced in both in vivo and ex vivo models [126, 127]. The antioxidant reinforcement at subcellular and cellular levels by the previously mentioned mechanisms would achieve an attenuation of oxidative stress injury secondary to anthracyclines. This was evidenced by a decrease in the production of mitochondrial H_2_O_2_ and in the peroxidation of membrane lipids expressed in lower concentrations of 8-isoprostanes in both the mitochondria as in cardiomyocyte membranes, which would also be associated with a reduction in microscopic scarring and tissue collagen [124, 125].

With respect to clinical evidence, a prospective study in 60 breast cancer patients on anthracyclines treatment showed that nebivolol had cardioprotective effects in the short term (6 months). The nebivolol group prevented diastolic dysfunction and had a lower reduction in global longitudinal strain compared with the control group [128, 129]. Therefore, basic and some clinical evidence supports a potential cardioprotective effect of nebivolol against AIC based on its pleiotropic properties beyond its beta-blocker effects.

### 3.5. Dexrazoxane: Topoisomerase 2*β* Target and Iron Chelators

Dexrazoxane is the only approved agent for the AIC prevention and is used intravenously in conjunction with the anthracycline to decrease the incidence of cardiomyopathy and congestive heart failure in a variety of cancer types in children and adults [130, 131].

In 2013, a meta-analysis showed a significant decrease in cardiac events for patients pretreated with dexrazoxane with no prior history of heart failure [90]. More recently, another new meta-analysis in patients with breast cancer treated with anthracyclines evaluated the efficacy of dexrazoxane of nine trials [132]. In this latter study, dexrazoxane reduced the risk of clinical heart failure and cardiac events in patients with anthracycline chemotherapy with or without trastuzumab and did not significantly impact cancer outcomes. However, the authors concluded that the quality of available evidence remains low, and further new randomized trials are warranted before a systematic implementation of this treatment in clinical practice [132]. In this sense, dexrazoxane treatment does not eliminate the risk of AIC, so it is necessary to continue clinical and cardiac function monitoring before and during therapy [130]. Moreover, dexrazoxane can be responsible for different adverse effects such as a reversible elevation of hepatic transaminases as well as some myelotoxicity (neutropenia and thrombocytopenia), limiting the dose given to the patient [133]. Dexrazoxane has two main mechanisms to ameliorate the AIC: (i) to chelate redox-active iron, thereby decreasing the formation of anthracycline-iron complexes preventing Fenton reaction and subsequently decreasing the ROS generation, which is harmful to the surrounding cardiac tissue [23, 130]; (ii) to act as a DNA topoisomerase II inhibitor, which happens to be the same target of the DNA Top2 anticancer agent (anthracyclines), antagonizing the formation of the Top2 cleavage complex and also rapidly degrading Top2*β* [134]. This does not induce harmful breaks in the double-strands of DNA in the heart as the anthracyclines [135]. However, given that other iron chelators have not shown a cardioprotective benefit after anthracycline treatments, it is possible that the primary protective mechanism would be through the inhibition of TOP 2*β* [23].

From the oxidative stress point of view, both mechanisms decrease ROS generation; first, directly for inhibition of Fenton reaction; second, indirectly because when it prevents the binding between anthracyclines and Top2*β*, it stops the next steps (mitochondrial dysfunction and ROS generation) [23, 130, 134].

Despite the plausible mechanisms and limited data, dexrazoxane is still not used routinely in clinical practice and is only FDA approved in the metastatic breast cancer population. Initially, there were concerns that dexrazoxane could attenuate the antitumor effects of anthracyclines and increase the occurrence of secondary malignancies [10], given its inhibition of Top2*α*, which is the anthracycline target in cancer cells [136]. However, dexrazoxane is currently considered not to be associated with a reduction in antitumor efficacy or survival or a relevant increased risk of second primary malignancies [132].

### 3.6. New Potential Interventions: Strategies Based on Non-ischemic Pharmacological Preconditioning: Omega 3 LCPUFA (DHA/EPA)

In recent years, other new strategies to prevent AIC are being evaluated. Some of these strategies proposed to prevent AIC are based on cardiac preconditioning, both ischemic and non-ischemic. Ischemic preconditioning has a broad preclinical base in cardiology, but it is usually complex to implement in cancer patients, and its efficacy would probably be limited. To our knowledge, currently, only one study (NCT02471885) is evaluating this type of strategy in AIC prevention [137]. However, for non-ischemic cardiac preconditioning, exercise has been proposed, based on preclinical evidence [138–140]. This kind of preconditioning can also be complex to apply in most cancer patients, but it is currently being tested by in a clinical trial (NCT02471053) [141].

Strategies based on non-ischemic pharmacological preconditioning have not been previously reported in clinical trials. However, a potential benefit of these interventions has been recently suggested by Serini et al., who hypothesized that n-3 Long-chain polyunsaturated fatty acids (LCPUFAs) could serve as cardio-protectors in AIC based on several preclinical models [142]. LCPUFAs have shown some evidence of having a role in the prevention and control of some cardiovascular diseases [143]. Specifically, EPA plus DHA has been shown to be efficacious in attenuating oxidative stress related to supraventricular arrhythmias in clinical trials [144–146]. Even though these effects have been associated with classical n-3 LCPUFAs properties, including anti-inflammatory activities, antiplatelet mechanisms, and biological membranes stabilization [147], more recent data suggest that indirect antioxidant properties could be more important [146]. It has been proposed that a high integration of n-3 LCPUFA into cardiomyocyte cell membranes would induce moderate lipid peroxidation, too weak to generate deleterious oxidative stress, but enough to activate the redox-sensitive transcription factor Nrf2. The activation of this factor upregulates antioxidant enzymes, thus, generating the pharmacological nonhypoxic myocardial preconditioning [148]. The activation of the Nrf2 pathway through n-3 LCPUFA and subsequent induction of antioxidant enzymes in cardiomyocytes have been described in both cellular [149] and preclinical models [150]. Regarding AIC, preclinical studies suggest that the Nrf2 pathway could play a role in physiological cardioprotection [151], as well as in the ability of n-3 LCPUFA to prevent doxorubicin-induced ROS production and the subsequent mitochondrial damage [152, 153]. Previously, a randomized controlled trial reported that n-3 LCPUFA nonhypoxic cardiac preconditioning was able to prevent postoperative atrial fibrillation trough enhancing endogenous heart antioxidant capacity [146].

Regarding the safety of high doses of n-3 LCPUFA in breast cancer patients treated with doxorubicin, there has been no clinical evidence of any harm or negative interactions. A randomized controlled trial reported no adverse effects in metastatic patients exposed to 1800 mg of DHA used as chemotherapy adjuvant, starting with a loading dose 7-10 days before initiating the first cycle of doxorubicin, and then maintaining the dose for five months [154]. Additionally, a 3-arm pilot double-blind placebo-controlled protocol was performed in patients with localized breast cancer undergoing first-time doxorubicin chemotherapy without finding any adverse effects in the n-3 LCPUFA arm [155]. In this arm, eleven patients were exposed to 2 g per day of n-3 LCPUFA (EPA + DHA) from 7 days before to 7 days after the first chemotherapy cycle, without any side effects associated with these fatty acids. Interestingly, the n-3 LCPUFA inhibited the expected NT-ProBNP plasma elevation after doxorubicin chemotherapy (48 hours), suggesting that the intervention was able to attenuate subclinical cardiotoxicity. Also, in the n-3 LCPUFA-treated group, there was a nonsignificant but lower echocardiography measured LVEF decline compared to double placebo patients at 10-12 months follow-up. The lack of statistical significance in this outcome is likely due to the small sample size and the high variability of echocardiography based LVEF measurements. Interestingly, despite these limitations, in the third arm, the eleven patients exposed to 12.5 mg of carvedilol every 12 hours showed a significantly lower reduction in LVEF at 10-12 months, compared to the double placebo group. Unexpectedly, in this study arm, carvedilol did not impact the levels of NT-ProBNP [155]. It is important to note that in this study, the population had a high prevalence of cardiovascular risk factors such as arterial hypertension (31%), dyslipidemia (22%), and smoking (42%) [155].

Currently, an ongoing clinical trial (CarDHA trial; ISRCTN69560410) in breast cancer patients receiving anthracyclines is designed to assess whether non-ischemic preconditioning with DHA plus carvedilol a week before the first chemotherapy cycle and, during 90 days after, would have a better capacity to limit subclinical AIC, compared with similar patients exposed to double placebo [156]. This study will evaluate any subclinical AIC manifestation in biomarkers, electrocardiographic alterations, LVEF by cardiac magnetic resonance, and global longitudinal strain by 2D echocardiography. Also, from a mechanistic point of view, the CarDHA trial is evaluating as secondary endpoint biomarkers of oxidative stress damage (plasma lipoperoxidation levels), as well as parameters of antioxidant balance (Erythrocyte Thiol Index (GSH/GSSG)). Therefore, this study will also enable the understanding of the impact of combined interventions (using as target two unrelated antioxidant pathways) on the inhibition or attenuation of the oxidative stress damage associated with AIC [156].

### 3.7. Combined Strategies: Classical (ACEi/ARBs plus Beta-Blockers) and New Combinations Targeting Different Unrelated Antioxidant Pathways (Omega 3 LCPUFA (DHA/EPA) plus Carvedilol)

Combined strategies have been previously poorly evaluated, and only two clinical trials have used dual interventions [109] [157]. However, the pathophysiological focus of these dual strategies has been the blockade of neuroendocrine systems (sympathetic nervous and the renin-angiotensin-aldosterone system) to try to modulate the remodeling process that occurs following a myocardial injury [109, 157].

In the OVERCOME (prevention of left-ventricular dysfunction with enalapril and carvedilol) trial, the combination of enalapril and carvedilol vs. no treatment was tested in 90 patients diagnosed with malignant hemopathies treated with anthracyclines. LVEF did not change in the intervention group but decreased significantly in controls after six months. Also, the intervention group had a lower incidence of final LVEF of <45 and heart failure [109].

Also, a combined beta-adrenergic and angiotensin blockade approach had been evaluated in one of the arms of the PRADA trial (Prevention of Cardiac Dysfunction during Adjuvant Breast Cancer Therapy) using a 2 × 2 factorial trial with metoprolol and candesartan during anthracyclines treatment [157]. This study showed that candesartan, but not metoprolol, can protect against an early decline in LVEF, assessed with cardiac MRI. It is also important to note that in this study they did not think there was a protective interaction between metoprolol and candesartan, due to combination was not better [157].

Unlike what was previously shown, new insights into the redox mechanism of damage in AIC allow consideration of new combined strategies. These new strategies with combined interventions based on the inhibition or attenuation of the oxidative stress injury associated with AIC by two unrelated antioxidant pathways could be more efficient than the potential for attenuation through treatments based on either one of the pathways alone.

An ongoing prospective randomized study by Carrasco et al. is investigating the potential role of a strategy based on two interventions with a focus on two different redox pathways for primary prevention of AIC [156].

Unlike other primary prevention protocols of AIC based on carvedilol alone, the CarDHA trial (ISRCTN69560410) is the first designed cardio-oncology study, based on using the antioxidant carvedilol properties associated with another antioxidant intervention as part of a dual therapeutic strategy focusing on attenuating the oxidative stress damage [156]. This clinical trial uses a sequential regimen. First, a DHA treatment is started one week before the anthracyclines to increase the antioxidant enzymatic activity in the myocardium, and then when the anthracycline is started, carvedilol is added, which provides direct antioxidant effects [156].

## 4. Conclusions

**Figure 1:**
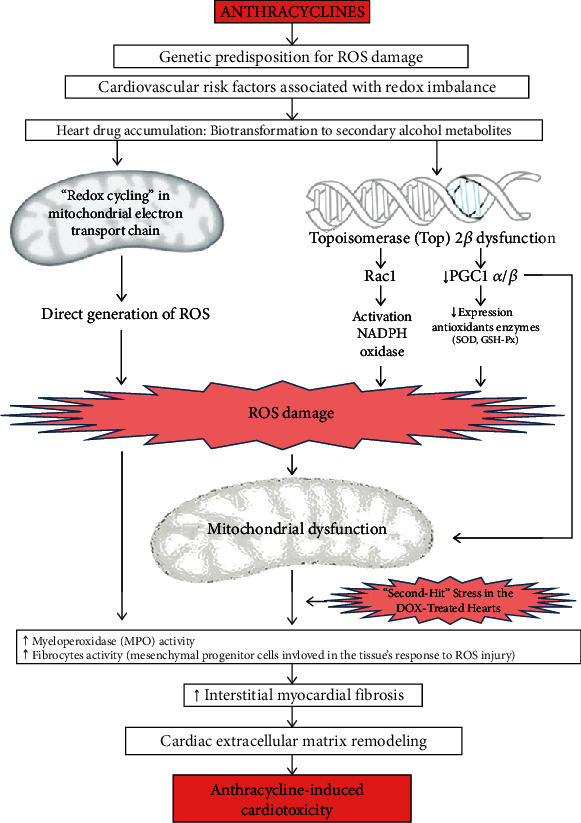
Mechanisms of anthracycline-induced cardiotoxicity and the role of oxidative stress. DOX: doxorubicin; GSH-Px: glutathione peroxidase; MPO: myeloperoxidase; PGC1 *α*/*β*: peroxisome proliferator-activated receptor-*γ* coactivator 1-*α* and 1-*β*; Rac1: a subunit of NADPH oxidase; ROS: reactive oxygen species; SOD: superoxide dismutase.

**Figure 2:**
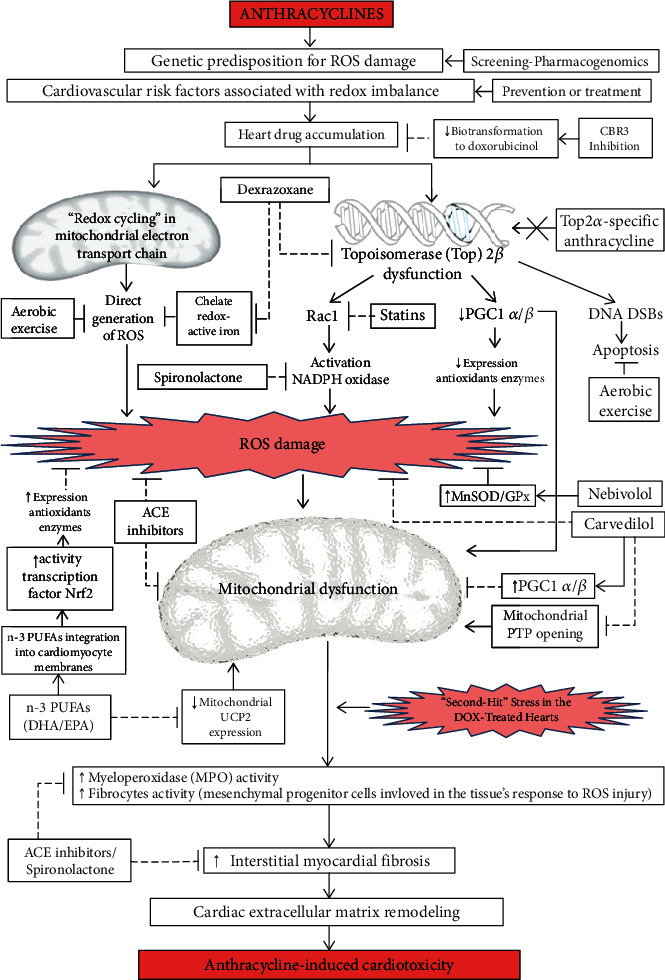
Potential cardioprotective therapies for anthracycline-induced cardiotoxicity. ACE: angiotensin-converting enzyme; CBR3: carbonyl reductase 3; DHA: docosahexaenoic acid; DNA DSBs: indicates deoxyribonucleic acid double-stranded breaks; EPA: eicosapentaenoic acid; GPx: glutathione peroxidase; MnSOD: Mn-superoxide dismutase; MPO: myeloperoxidase; n-3 PUFAs: n-3 polyunsaturated fatty acids; Nrf2: nuclear factor erythroid 2-related factor 2; PGC1 *α*/*β*: peroxisome proliferator-activated receptor-*γ* coactivator 1-*α*, and 1-*β*; PTP: permeability transition pores; Rac1: a subunit of NADPH oxidase; ROS: reactive oxygen species; Top2*β*: topoisomerase 2*β*; UCP2: mitochondrial uncoupling protein 2.

The multiple mechanisms proposed for anthracycline cardiotoxicity could be grouped under the umbrella of a unifying downstream mechanism: “oxidative stress” (Figure 1). Therefore, most of the interventions that could be beneficial in the primary prevention of AIC have potential antioxidant effects as a common theme (Figure 2).

Due to this crucial role of oxidative stress in AIC, the screening of cardiovascular risk factors associated with oxidative imbalance is essential to identify the subgroup of patients that could benefit the most from a primary prevention strategy with antioxidants interventions. Also, the development of a genetic approach to identify some polymorphisms in genes related to anthracyclines biotransformation, antioxidant defences, or ROS generation could help to find patients with an increased risk of AIC.

Although in the future, it may be attractive to develop new preventive strategies for AIC, focused on targets such as topoisomerase 2*β* or the biotransformation of anthracyclines, the imperative need to have preventive interventions for AIC in the short term promotes continuing the evaluation of strategies that reduce oxidative stress through the use of drugs already available. The therapeutic interventions that have been clinically evaluated, such as ACE inhibitors, aldosterone antagonists, carvedilol, and nebivolol, contribute to decrease the severity of redox imbalance by different mechanisms and could also have a role in reducing the impact caused by “second hits” (Figure 2). New strategies involving non-ischemic cardiac preconditioning are being evaluated as preventative options for AIC due to their capacity of attenuating oxidative stress. Also, the role of dual strategies based on combined interventions targeting different redox pathways for the primary prevention of AIC is being evaluated by new clinical trials.

This review also has some limitations. First, the evidence for most antioxidant interventions is still limited, and mostly all studies have small sample sizes. Second, although some interventions could theoretically prevent AIC through their antioxidant properties (such as carvedilol), most of the studies only evaluated clinical endpoints but not oxidative stress parameters to provide better mechanistic evidence.

Finally, despite the multiple new advances in knowledge of anthracycline-induced cardiotoxicity, oxidative stress remains one of the main therapeutic targets for cardioprotection. Therefore, further studies are needed with clinical interventions focused on the reduction of oxidative stress.
